# Integrating charge mobility, stability and stretchability within conjugated polymer films for stretchable multifunctional sensors

**DOI:** 10.1038/s41467-022-30361-0

**Published:** 2022-05-18

**Authors:** Sung Yun Son, Giwon Lee, Hongyu Wang, Stephanie Samson, Qingshan Wei, Yong Zhu, Wei You

**Affiliations:** 1 Department of Chemistry, University of North Carolina at Chapel Hill, Chapel Hill, NC 27599 USA; 2 Department of Chemistry, Kwangwoon University, Seoul, 01897 Republic of Korea; 3 Department of Chemical and Biomolecular Engineering, North Carolina State University, Raleigh, NC 27695 USA; 4 Department of Mechanical and Aerospace Engineering, North Carolina State University, Raleigh, NC 27695 USA; 5 Department of Applied Physical Sciences, University of North Carolina at Chapel Hill, Chapel Hill, NC 27599 USA

**Keywords:** Polymers, Organic molecules in materials science, Polymers

## Abstract

Conjugated polymers (CPs) are promising semiconductors for intrinsically stretchable electronic devices. Ideally, such CPs should exhibit high charge mobility, excellent stability, and high stretchability. However, converging all these desirable properties in CPs has not been achieved via molecular design and/or device engineering. This work details the design, synthesis and characterization of a random polythiophene (RP-T50) containing ~50 mol% of thiophene units with a thermocleavable tertiary ester side chain and ~50 mol% of unsubstituted thiophene units, which, upon thermocleavage of alkyl chains, shows significant improvement of charge mobility and stability. Thermal annealing a RP-T50 film coated on a stretchable polydimethylsiloxane substrate spontaneously generates wrinkling in the polymer film, which effectively enhances the stretchability of the polymer film. The wrinkled RP-T50-based stretchable sensors can effectively detect humidity, ethanol, temperature and light even under 50% uniaxial and 30% biaxial strains. Our discoveries offer new design rationale of strategically applying CPs to intrinsically stretchable electronic systems.

## Abstract (web summary)

Conjugated polymers are promising semiconductors for stretchable electronic devices but combining important properties such as high charge mobility, stability and stretchability remains challenging. Here, the authors demonstrate the synthesis of a thiophene based semiconducting polymer with cleavable side chains which shows significant improvement of charge mobility, stability and stretchability.

## Introduction

Conjugated polymers (CPs) need side chains to ensure their solution processability, a key prerequisite to enable low-cost large-area production of functional devices with CPs as the core component. However, these side chains often lead to a low glass transition temperature (*T*_*g*_) of CPs, resulting in morphological instability of CP-based thin films at elevated temperatures^1^. Furthermore, these side chains (typically hydrocarbons) are prone to photo-oxidation and consequently, photo-induced degradation of CP-based thin films^2^. To address these issues, thermocleavable side chains (TCSs) have been actively explored in recent years. This is because CPs, upon removing TCSs by heating, could achieve much increased *T*_g_ and become less influenced by photo-oxidation^3^. For example, we have recently shown that polythiophenes with TCSs can achieve photovoltaic efficiencies of 1.5% and maintain 90% of its initial performance after 24 h under harsh testing conditions (150 °C, in N_2_ and under ambient light), largely due to the much improved *T*_g_ after removing TCSs^4^.

However, one significant challenge is the often observed lower device performances (e.g., lower charge carrier mobility) after the cleavage of these TCSs when compared with ‘identical’ polymers with conventional alkyl side chains^5,6^. This is because TCSs typically contain thermally labile functionalities such as esters, amides, or carbonates linked with bulky (often tertiary) alkyl chains^3^ (to ensure the thermocleavage of alkyl chains at a relatively low temperature and the solubility of CPs^7^). These sterically demanding side chains result in enhanced backbone torsion and reduced π–π stacking between adjacent conjugated backbones, both of which can seriously impair the charge transport before the thermocleavage. While thermally removing these bulky side chains could alleviate these steric-associated impacts and improve the charge transport, the applied thermal-energy-induced movement of CPs, accompanied by the generation of often volatile species (from these cleaved side chains), can lead to uncontrolled morphological changes. These undesirable morphological changes are kinetically trapped after TCSs are removed (i.e., when such CP-based thin films are vitrified) and become a significant issue for reaching high device performance. For example, serious phase separation in the case of bulk heterojunction blends would lead to lower efficiency in such polymer-based solar cells^8^.

Interestingly, in earlier work, Son et al. showed that random polythiophenes with low crystallinity but localized aggregates formed via π–π stacking were able to achieve higher charge carrier mobility than that of regioregular poly(3-hexylthiophene) (P3HT) with high crystallinity^9^. They introduced unsubstituted thiophene units into P3HT in a random manner, and the reduced density of hexyl side chains resulted in enhanced backbone planarity and hence the degree of π–π stacking, thereby forming localized aggregates which are believed to account for the higher mobility. We envision that introducing these unsubstituted thiophene units into our polythiophenes with TCSs could enhance the backbone planarity and the degree of π–π stacking, and also mitigate uncontrolled morphological changes (due to less TCSs on the backbone), all of which could lead to high mobility after thermocleavage of side chains.

**Fig. 1:**
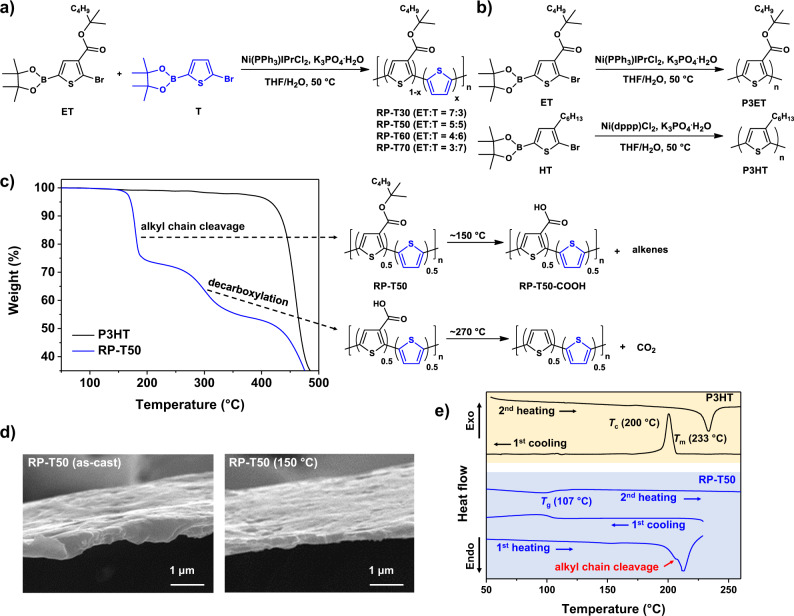
Synthesis and characterization of polymers. Synthetic schemes for RP-T series (**a**), and P3ET and P3HT (**b**). **c** TGA thermograms of P3HT and RP-T50, and schemes of alkyl chain cleavage and decarboxylation of RP-T50. The alkyl chains decompose into alkenes and leave RP-T50-COOH behind after the alkyl chain cleavage. **d** SEM images of as-cast and 150 °C annealed RP-T50 films. **e** DSC thermograms of P3HT and RP-T50.

To experimentally investigate this hypothesis, we synthesized a series of polythiophenes by copolymerizing the thiophene monomer bearing a thermocleavable tertiary ester side chain (ET) and the unsubstituted thiophene monomer (T) at four different feed ratios via chain-growth Suzuki polycondensation^10^ (Fig. 1a). Pleasingly, we found that RP-T50, where half of the repeat units are ET and the other half are T, showed a great improvement in charge mobility after thermocleavage of alkyl chains (from 7.45 × 10^−6^ to 7.46 ± 4.67× 10^−3^ cm^2^/V·s, measured by space-charge-limited current (SCLC) method). Importantly, this mobility is significantly higher than that of P3HT (9.18 ± 5.44 × 10^−4^ cm^2^/V·s) in this study. In addition, RP-T50 films (after thermocleavage of alkyl chains) exhibited excellent stability against chloroform wash and under harsh conditions (100 °C, in air and under continuous ambient light for 24 h).

Further characterization of RP-T50 via differential scanning calorimetry (DSC) revealed that RP-T50 exhibited a high *T*_g_ (~107 °C) after removing alkyl chains. Moreover, we found that the Young’s modulus of a RP-T50 film significantly increased by a factor of ~1000 upon removal of the alkyl chains, suggesting that the RP-T50 film became rigid after cleavage of alkyl chains and thus would not be suitable for stretchable electronics applications. However, the rigid nature of RP-T50 film (after thermocleavage of alkyl chains) allowed us to create wrinkled structures in RP-T50 films by using stretchable polydimethylsiloxane (PDMS) substrates^11^. Importantly, the ‘wrinkled’ RP-T50 film showed significantly enhanced stretchability compared to that of ‘flat’ RP-T50 film. These findings suggest that simultaneous improvement in charge mobility, stability, and stretchability – which typically cannot be fulfilled with one particular CP – can be offered with RP-T50 with strategic chemical design and device engineering. Finally, we applied such wrinkled RP-T50 films (after thermocleavage of alkyl chains) as stretchable organic sensors to take advantage of their high charge mobility, stability, and stretchability. The wrinkled RP-T50 film-based sensors exhibited much improved sensing behavior toward various external stimuli such as humidity, ethanol, temperature, and light compared to P3HT-based sensors. Furthermore, the wrinkled RP-T50 film-based sensors were able to maintain the sensing performance under 50% uniaxial strain and 30% biaxial strain.

## Results and discussion

### Synthesis and characterization

We synthesized four different random polythiophenes via chain-growth Suzuki polycondensation by varying the monomer feed ratio between T and ET (30, 50, 60, and 70 mol% T), generating RP-T30, RP-T50, RP-T60, and RP-T70, respectively (Fig. 1a; detailed synthetic procedures are provided in the Supplementary Information). Not surprisingly, as the mol% of T increases, the solubility of the resulting polythiophene decreases. While RP-T30 and RP-T50 have good solubility in chloroform (>15 mg/ml), both RP-T60 and RP-T70 are only partially soluble in the solvent (<1 mg/ml). We hypothesize that more unsubstituted thiophene units (i.e., T) in the backbone would be beneficial to enhancing the backbone planarity and/or the degree of π–π stacking (before thermocleavage of alkyl chains), and also to mitigating morphological change during thermocleavage. As such, we decided to focus on RP-T50 to investigate charge mobility and stability before/after removal of the alkyl chains. For comparison, we synthesized regioregular P3HT (100 mol% hexyl side chains) and P3ET (100 mol% ET that has the thermocleavable ester side chain) via chain-growth Suzuki polycondensation as well (Fig. 1b). Number average molecular weights (*M*_n_) and dispersities (*Đ*) of RP-T50, P3HT, and P3ET were determined by gel permeation chromatography (GPC) using polystyrene standards as the calibration (RP-T50: 64,000 (*Đ*: 1.28), P3HT: 37,700 (*Đ*: 1.29), and P3ET: 56,700 (*Đ*: 1.55)).

Characterization of RP-T50 using ^1^H NMR spectroscopy revealed that the actual molar ratio of unsubstituted thiophene unit was 46 mol% (Supplementary Fig. 1), matching well with the monomer feed ratio of T (50 mol%). Thermogravimetric analysis (TGA) of RP-T50 at a heating rate of 10 °C/min disclosed that the alkyl chains of the tertiary ester side chains were cleaved at 150 °C and left RP–T50–COOH behind, followed by the occurrence of decarboxylation at around 270 °C (Fig. 1c). The experimental weight loss for the alkyl chain cleavage (28.8%) is slightly lower than the theoretical value (34%), whereas the experimental weight loss for the decarboxylation (17.9%) is slightly higher than the theoretical value (15.2%). Nevertheless, the total weight loss observed (46.7%) is well matched with the theoretical value for the combined weight loss of both steps (49.2%). This suggests that there could be some alkyl chains uncleaved or cleaved but trapped in the vitrified film that were removed at higher temperatures. Further increasing the temperature eventually degraded RP-T50 at ~425 °C and P3HT at a similar temperature (~415 °C). All these thermal results are consistent with those of closely related polythiophenes containing identical TCS units in our earlier work^4^. In our earlier work, we demonstrated that removing the alkyl chains at relatively low temperature (e.g., 150 °C) is sufficient to achieve much improved morphological stability, and further ramping up the annealing temperature (e.g., >270 °C) to remove carboxylic acids does not offer additional benefits^4^. Here, we decided to applied similar thermal annealing (e.g., 150 °C for 12 h or 200 °C for 30 min) to remove only the alkyl chains in RP-T50, affording RP–T50–COOH as the key material in this study. Moreover, higher temperature (>270 °C) would be too harsh when flexible or stretchable substrates are used (e.g., indium tin oxide-coated polyethylene terephthalate or polyethylene naphthalate)^12^.

The alkyl chain cleavage in a RP-T50 film was further confirmed via infrared (IR) spectroscopy (Supplementary Fig. 2). Upon annealing at 150 °C up to 12 h, the ester C=O stretch (1706 cm^−1^) and alkane C−H stretch (3000–2800 cm^−1^) peaks in the as-cast RP-T50 film disappear, while a new carboxylic acid C=O stretch peak (1685 cm^−1^) and O−H stretch band (3500–2500 cm^−1^) arise, indicating the presence of carboxylic acid. The broad carboxylic acid O−H stretch band is attributed to hydrogen bonding between carboxylic acids on the backbones^13^. It is worth noting that the carboxylic acid C=O stretch peak started to appear from the RP-T50 film annealed at 150 °C for 3 h. In addition, the carboxylic acid C=O stretch peak began to be more dominant than the ester C=O stretch peak in the RP-T50 film after annealed at 150 °C for 5 h, indicating significant amount of the alkyl chains were cleaved after 5 h at 150 °C. It is known that the film thickness would be reduced upon removal of side chains since polymer backbones can pack more closely in the absence of side chains^7^. Indeed, the thickness of the RP-T50 film noticeably decreased after thermal annealing at 150 °C for 12 h (Fig. 1d). For example, two different thicknesses (496 and 915 nm, before thermocleavage) decreased to ~56 % of the initial values. Using the film thicknesses measured and the theoretical weight of RP-T50 after alkyl chain cleavage (66% of the initial weight), we calculated the normalized film density after alkyl chain cleavage (Supplementary Table 1). The normalized film density increased to 1.18 times the initial value after thermal annealing, suggesting that the interspacing of RP-T50 polymer chains is reduced due to the thermal cleavage of alkyl chains.

Further characterization of these polymers via DSC revealed more insights. P3HT showed distinct crystallization and melting peaks in the first cooling curve and the second heating curve, respectively, indicative of its semi-crystalline nature (Fig. 1e). On the other hand, RP-T50 showed alkyl chain cleavage in the first heating curve and a glass transition at around 107 °C in both the first cooling and the second heating curves, suggesting that RP-T50 has a very low crystallinity and should be rigid (i.e., ‘glassy’) at room temperature after alkyl chain cleavage. We further investigated changes in the rigidity of RP-T50 films upon alkyl chain cleavage by measuring the Young’s moduli of RP-T50 films before and after thermal annealing at 150 °C for 12 h. While the film-on water^14,15^ and buckling-on-elastomer^16^ methods have been widely employed to acquire Young’s modulus data of thin films, we chose the buckling-based metrology method due to its simple and facile measuring process. With this method, we repeated three times with three individual samples for consistency of our results (detailed procedures are provided in the Supplementary Information). The Young’s modulus of the RP-T50 film was found to increase by a factor of ~1000 after alkyl chain cleavage (from 2.57–18.7 MPa to 2.64–4.52 GPa), clearly supporting that the rigidity of RP-T50 films significantly increased upon removal of alkyl chains. The enhanced Young’s modulus of RP-T50 upon removal of alkyl chains (i.e., RP–T50–COOH) can be attributed to the increase in the volume fraction of rigid conjugated backbones and consequently enhanced π–π stacking between backbones^17^. In addition, the hydrogen bonding between carboxylic acids on the backbones (as observed using IR spectroscopy) could also contribute to the increase in Young’s modulus.

### Optical properties and microstructures

**Fig. 2:**
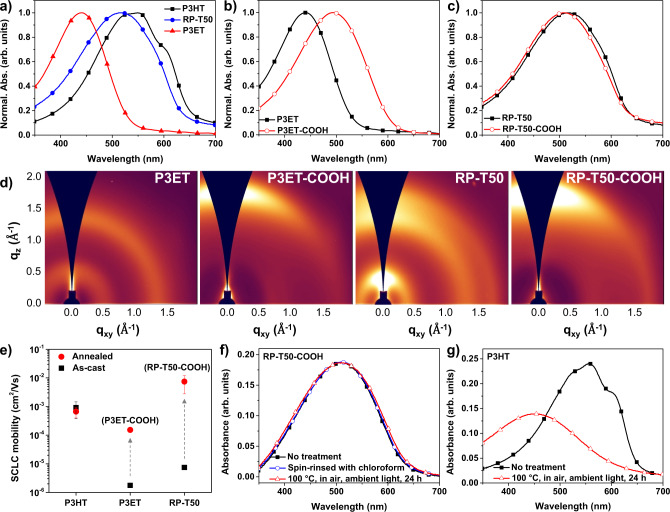
Optical properties, microstructures, SCLC mobility and stability test. **a** Normalized UV–vis absorption spectra of as-cast P3HT, RP-T50 and P3ET films. Normalized UV–vis absorption spectra of P3ET and P3ET–COOH films (**b**) and RP-T50 and RP–T50–COOH films (**c**). **d** GIWAXS patterns of P3ET, P3ET–COOH, RP-T50 and RP–T50–COOH films. **e** SCLC mobilities of P3HT, RP-T50, and P3ET before and after annealing at 150 °C for 12 h. Error bars represent ± s.d. (*n* = 2–4). **f** UV–vis absorption spectra of RP–T50–COOH before and after stability test (spin-rinsed with chloroform only or heated at 100 °C, in air and under continuous ambient light for 24 h after spin-rinsed with chloroform). **g** UV–vis absorption spectra of P3HT before and after stability test (heated at 100 °C, in air and under continuous ambient light for 24 h).

To investigate the chain conformation and packing mode of RP-T50, the UV–vis absorption spectrum of an as-cast RP-T50 film was compared with those of as-cast P3HT and P3ET films (Fig. 2a). As expected, the as-cast P3HT film shows an absorption maximum at 550 nm and a distinct shoulder peak around 600 nm. By contrast, the spectrum of P3ET is considerably blue-shifted compared to that of P3HT and does not have any noticeable shoulder peaks. This indicates that the rather bulky tertiary ester side chains on P3ET lower the backbone planarity and also sterically hinder π–π stacking between neighboring conjugated backbones^9,18,19^. Interestingly, the absorption of RP-T50 is considerably red-shifted compared to that of P3ET, yet slightly blue-shifted compared to that of P3HT. In addition, a subtle shoulder peak around 570 nm can be observed for the as-cast RP-T50 film. These results suggest that the unsubstituted thiophene units in RP-T50 (46 mol%) are able to alleviate steric hindrance between the tertiary ester side chains, leading to enhanced backbone planarity and π–π stacking^9,20^. We further investigated the absorption spectra of P3ET and RP-T50 films after annealing them at 200 °C for 30 min to remove the alkyl chains (i.e., an accelerated process to thermally remove alkyl chains). While such annealed P3ET film (i.e., P3ET–COOH film) displays a distinct change (i.e., red-shift by 54 nm) when compared with its as-cast film (Fig. 2b), the annealed RP-T50 film (i.e., RP–T50–COOH film) does not show significant differences in its absorption when compared with the same film pre-annealed (Fig. 2c). These results indicate that removing alky chains in P3ET films incurred considerable morphological changes; by contrast, thermally removing alkyl chains in RP-T50 films appears to cause little morphological changes. These observations can be explained by the much reduced amount of sterically bulky TCSs in the case of RP-T50 (~50 mol%, vs. 100 mol% for P3ET) and the presence of unsubstituted thiophene (T), both of which could largely maintain the backbone planarity and minimize the steric hindrance among chains. In addition, the absorption of RP–T50–COOH is slightly red-shifted compared to that of P3ET–COOH, indicating RP–T50–COOH, with the help of 50 mol% unsubstituted thiophene (T), exhibits higher backbone planarity than P3ET–COOH (Supplementary Fig. 3).

To further compare morphological changes in P3ET and RP-T50 upon thermocleavage of the alkyl chains, we investigated P3ET, P3ET-COOH (i.e., P3ET annealed at 150 °C for 12 h), RP-T50 and RP–T50–COOH (i.e., RP-T50 annealed at 150 °C for 12 h) films using grazing incidence wide-angle X-ray scattering (GIWAXS) (Fig. 2d). The as-cast P3ET film does not show any noticeable scattering peaks (it should be noted that a weak (010) peak was observed at 1.37 Å^−1^ in the out-of-plane spectrum extracted from the GIWAXS pattern (Supplementary Fig. 4)), indicating the near amorphous nature of the as-case P3ET film, likely because the bulky tertiary ester side chains prevent close and regular polymer chain packing. However, after thermocleavage of alkyl chains, the P3ET–COOH film shows a distinct out-of-plane (010) peak at 1.71 Å^−1^. This suggests that upon removal of the alkyl chains, the formation of out-of-plane π–π stacking was facilitated. On the other hand, the as-cast RP-T50 film shows one (100) peak and two (010) peaks along the out-of-plane direction, indicative of much enhanced polymer chain ordering; this difference in packing/ordering of polymers between as-cast P3ET and RP-T50 film clearly demonstrates that the unsubstituted thiophene units incorporated in RP-T50 can effectively mitigate the steric hindrance of the TCSs. Interestingly, it was found that the two (010) peaks in the GIWAXS pattern of the RP-T50 film appeared at similar q_z_ values (1.37 and 1.71 Å^−1^) as for P3ET (1.37 Å^−1^) and P3ET-COOH (1.71 Å^−1^) (Supplementary Fig. 4), implying the existence of two different π–π stacking modes which can be attributed to the two structurally very different thiophenes units in RP-T50 (i.e., ET and T)^9^. After thermocleavage of alkyl chains, only one out-of-plane (010) peak (1.71 Å^−1^) remains in the RP–T50–COOH film since the peak at 1.37 Å^−1^ (due to sterics of TCSs) would disappear and merge into the peat at 1.71 Å^−1^. These results suggest that unlike P3ET, the as-cast RP-T50 film already has strong π–π stacking/ordering of polymer chains before removing alkyl chains and was able to sustain the stacking during thermocleavage.

Furthermore, we have quantified π–π stacking in P3ET–COOH and RP–T50–COOH films by constructing geometrically corrected pole figures for the (010) π–π stacking peak from the GIWAXS patterns and comparing the total integrated areas between P3ET–COOH and RP–T50–COOH films. The geometrically corrected pole figures were constructed according to procedures discussed in previous works (Supplementary Fig. 5)^21,22^. The total integrated areas for RP–T50–COOH is ~1.95 times larger than that for P3ET–COOH, suggesting the degree of π–π stacking in the RP–T50–COOH film is significantly higher.

### Charge mobility and morphological stability

We next evaluated charge mobilities of P3HT, RP-T50, and P3ET before and after thermal annealing (150 °C, 12 h) via the SCLC method. The current density–voltage curves are shown in Supplementary Fig. 6 and the charge mobilities are summarized in Supplementary Table 2. The SCLC mobility of P3HT slightly decreased after thermal annealing (from 9.18 ± 5.44 × 10^−4^ to 6.67 ± 2.67 × 10^−4^ cm^2^/V·s) (Fig. 2e); by contrast, the mobilities of RP-T50 and P3ET were significantly improved after thermal annealing: from 7.45 × 10^−6^ to 7.46 ± 4.67 × 10^−3^ cm^2^/V·s for RP-T50, and from 1.76 ± 0.01 × 10^−6^ to 1.53 ± 0.29 × 10^−4^ for P3ET. We ascribe this improvement in mobility to the considerably enhanced π–π stacking upon removal of the alkyl chains (Fig. 2d). Importantly, the SCLC mobility of the annealed RP-T50 (i.e., RP-T50-COOH) was improved by three orders of magnitude, even much higher than that of P3HT. To the best of our knowledge, this is the first observation that a CP bearing TCS shows higher mobility than that of its parent polymer. To provide further evidence on the key role of incorporating unsubstituted thiophene (T) units into the polymer backbone to achieve such dramatic increase of charge mobility after thermocleavage of alkyl chains, we measured SCLC mobility of a polythiophene containing ~50 mol% TCSs and ~50 mol% hexyl side chains (RP-TCS50, *M*_n_: 54,500 (*Đ*: 1.91)) before and after thermal annealing at 150 °C for 12 h. While the SCLC mobility of RP-TCS50 was also significantly improved after thermal annealing, the value (6.87 ± 1.97 × 10^−5^ cm^2^/V·s) is still considerably lower than that of RP–T50–COOH (Supplementary Fig. 7).

Finally, we investigated the morphological stability of RP–T50–COOH (i.e., RP-T50 annealed at 200 °C for 30 min). Experimentally, we monitored changes in the UV–vis absorption spectrum of RP–T50–COOH film after it was spin-rinsed with chloroform (see Supplementary Fig. 8a for the schematics of experimental setup) or tested under the chosen testing conditions (100 °C, in air and under continuous ambient light for 24 h). For comparison, similar experiments were also conducted on as-cast P3HT films. The absorption spectrum of the RP–T50–COOH film remained unchanged even after the film was spin-rinsed with chloroform (Fig. 2f), indicating that RP-T50 became insoluble after the solubilizing alkyl chains were thermally removed. Further subjecting the post-rinsing RP–T50–COOH film to the rather harsh stability testing did not appear to affect the integrity of the film, evidenced by no distinct changes in the absorption spectrum after the stability test (Fig. 2f). By contrast, the P3HT film was almost completely removed after being spin-rinsed with chloroform (Supplementary Fig. 8b). In addition, the spectrum of the other P3HT film that was kept under the stability testing condition showed significant blue-shift and reduced absorbance (Fig. 2g). These results clearly showed that RP–T50–COOH exhibits much higher morphological stability than P3HT.

### Spontaneous wrinkle formation in RP-T50 film by thermal annealing

While the much enhanced charge mobility and excellent morphological stability of the RP–T50–COOH film are exciting for device applications, the rigid nature of RP–T50–COOH film would not be desirable for applications such as stretchable electronics. In searching for possible strategies, we noted that introducing wrinkling structures in rigid films such as metal foils can effectively enhance the stretchability of such films^23^. However, the procedure for the fabrication of wrinkling structures typically involves rather complicated steps, e.g., pre-straining the substrate then depositing a thin film on top, followed by releasing the pre-strained substrate together with the deposited film^23,24^.

**Fig. 3:**
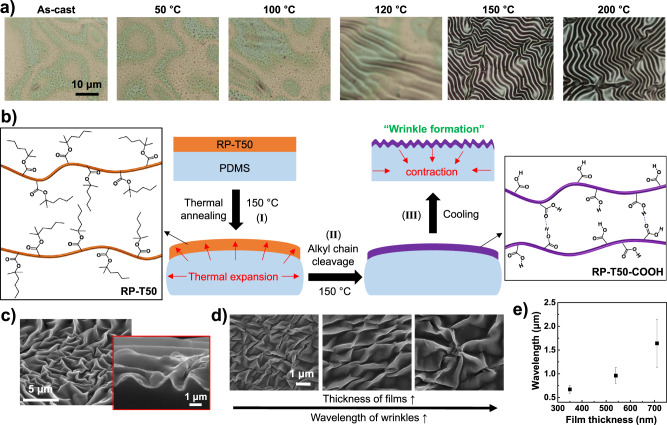
Mechanism of the wrinkling phenomena on RP-T50 film. **a** Optical microscopic images of RP-T50 films coated on a PDMS substrate before (as-cast) and after annealing at various temperatures. **b** Proposed mechanism of spontaneous formation of wrinkled structure in a RP-T50 film upon thermal annealing. **c** SEM image of the surface of the RP–T50–COOH film (annealed at 150 °C) on PDMS. Inset: the magnified image of the wrinkled surface with a cross-sectional view. **d** SEM images of RP–T50–COOH films with three different thicknesses. As the thickness of the film increases, the wavelength of wrinkles is enhanced. **e** Plot of the wavelength of wrinkles in RP–T50–COOH films as a function of film thickness. The thicknesses were measured after annealing RP-T50 films at 150 °C for 12 h. Error bars represent mean ± s.d., with *n* = 3.

Interestingly, we found spontaneous wrinkling of a RP-T50 film on a PDMS substrate after the alkyl chain cleavage. Experimentally, we prepared a pristine RP-T50 film on an UV–ozone treated PDMS substrate by drop-casting the RP-T50 solution in chloroform (10 mg/ml), annealed the RP-T50/PDMS at a predetermined temperature (50, 100, 120, 150, and 200 °C) for 12 h, and finally cooled the RP-T50/PDMS to room temperature under ambient condition. We observed the formation of wrinkles when the annealing temperature was above 120 °C (Fig. 3a), the temperature at which the alkyl chain cleavage started to occur^4^. More wrinkles can be clearly observed after annealing at higher temperatures (150 and 200 °C). These results, together with the modulus data, suggest a plausible mechanism of wrinkle formation as follows. At the beginning of thermal annealing at 150 °C, PDMS is thermally expanded more than RP-T50, leading to tensile stress in the RP-T50 film (step (I) in Fig. 3b) because no alkyl chain cleavage occurs and the RP-T50 film has a low Young’s modulus (2.57–18.7 MPa). As time progresses (e.g., ~3 h), alkyl chain cleavage and morphological changes starts, and continues until most of alkyl chains are removed (step (II) in Fig. 3b); RP–T50–COOH forms with a decrease in the film thickness and increase in its Young’s modulus (2.64–4.52 GPa). During this process, the tensile stress in the film might be released because heat-induced high mobility of polymer chains can promote stress relaxation^25^. Upon cooling to room temperature, thermal contraction of PDMS leads to compressive stress in the RP-T50-COOH film, which drives the formation of the wrinkles (step (III) in Fig. 3b). Further characterization of the RP–T50–COOH film on a PDMS substrate using scanning electron microscopy (SEM) revealed that the wrinkles follow the herringbone type of patterns, typical of biaxial wrinkling^26,27^; no delamination was observed between the RP–T50–COOH film and the PDMS substrate (Fig. 3c). It should be noted that wrinkles did not form in P3HT films coated on PDMS despite annealed under the same condition used for RP-T50 films.

In addition, we can control the wavelength of wrinkles in RP–T50–COOH films by varying the thickness of the pre-annealed RP-T50 film (Fig. 3d); the wavelength was measured by calculating the average from random wrinkles^28^. According to the theory of wrinkling phenomena, the wavelength (λ) from one-dimensional wrinkle can be related with the film thickness (h) and plane-strain moduli ($\bar{E}$) via ${{{{{\rm{\lambda }}}}}}=2{{{{{\rm{\pi }}}}}}{{{{{\rm{h}}}}}}{(\frac{{\bar{E}}_{f}}{3{\bar{E}}_{s}})}^{1/3}$, where the subscripts s and f represent substrate and film, respectively. Moreover, the minimum energy state for the case of herringbone wrinkles has undulation width, which is very close to the wavelength of the one-dimensional mode^26^. The equation shows that the wavelength is proportional to the thickness of the film, which is consistent with our experimental results (Fig. 3e). For example, as the thickness of RP–T50–COOH film increases from 350 to 720 nm, the wavelength increases from 0.67 to 1.66 μm.

### Stretchability of wrinkled RP-T50-COOH films

**Fig. 4:**
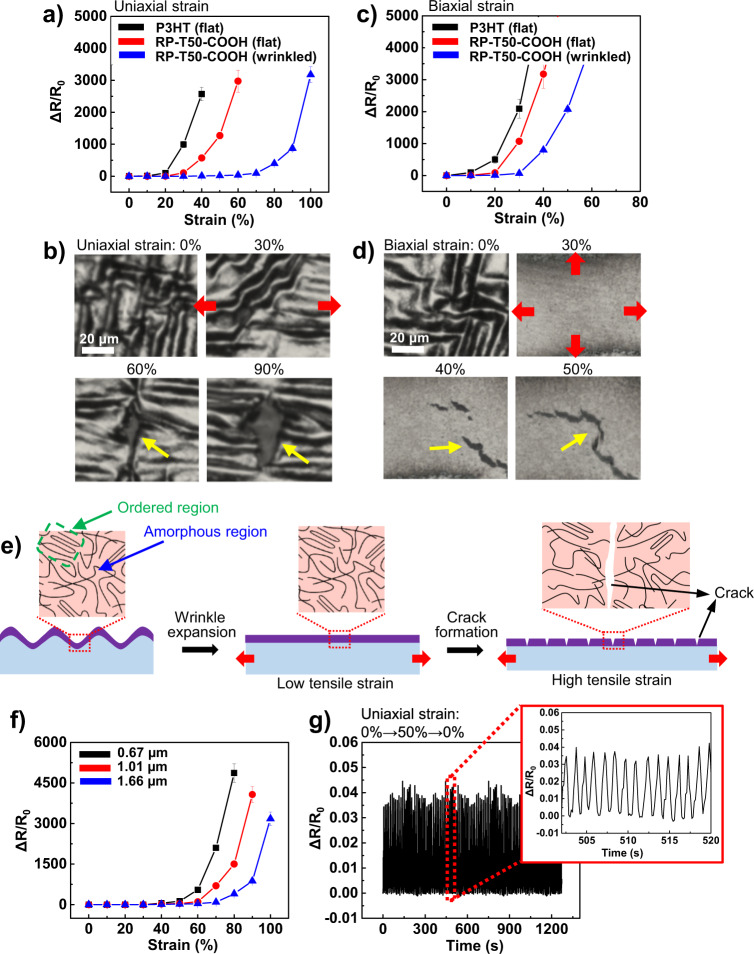
Stretchability of the wrinkled polymer thin film. Electrical resistance changes under uniaxial strain (**a**) and biaxial strain (**c**). Sequential optical microscope images of the wrinkle deformation with uniaxial strain (**b**) and biaxial strain (**d**) using wrinkled RP-T50-COOH samples. Yellow arrows indicate crack formation. **e** Schematic illustration of the deforming and crack formation mechanism in the wrinkled RP-T50-COOH film under uniaxial strain. **f** Resistance change versus tensile strain depending on the average wavelength of wrinkles using wrinkled RP-T50-COOH film. **g** Uniaxial cyclic test to confirm the mechanical robustness for wrinkled RP-T5-COOH film. Inset: the magnified view of the graph. Error bars in **a**, **c** and **f** represent mean ± s.d., with *n* = 3.

To evaluate the stretchability, we measured the electrical resistance changes of flat P3HT, flat RP–T50–COOH and wrinkled RP–T50–COOH films coated on a PDMS substrate under uniaxial and biaxial strains. While flat P3HT and wrinkled RP–T50–COOH films were prepared by thermally annealing the films coated on a PDMS substrate at 150 °C for 12 h and subsequently cooling them to room temperature under ambient condition, the flat RP–T50–COOH film was prepared on octadecyltrichlorosilane (OTS) treated glass slide, followed by thermal annealing and cooling the film under the same condition, then transferring the film to a PDMS substrate. As shown in Fig. 4a, the wrinkled RP–T50-COOH exhibits the lowest resistance change even under 70% of the uniaxial tensile strain. By contrast, rapid resistance increase was observed at much lower tensile strain for other polymer films, 20% for the flat P3HT film and 30% for the flat RP–T50–COOH film. We applied an optical microscope to observe the deformation of the wrinkled RP–T50–COOH film under the uniaxial tensile strain (Fig. 4b). Before 60% tensile strain, the wrinkles are elongated along the strain direction. However, when the strain reaches 60%, newly formed cracks on the film are clearly visible, which apparently widen as the strain increases to 90%. These cracks apparently account for the rapidly increased electrical resistance of the film as the applied strain surpasses 60%. Furthermore, when biaxial stretching was applied to all these films, the wrinkled RP–T50–COOH film again has the highest stretchability among all the studied films before significant resistance increase kicks in (Fig. 4c). However, the absolute value of stretchability in biaxial strain is lower than that obtained in uniaxial strain. For example, the critical strain of crack initiation appears at 30% biaxial strain vs. at 60% uniaxial strain (Fig. 4d). This result is originated from the fact that biaxial strain suppresses the Poisson’s effect under uniaxial strain. Briefly, while uniaxial strain has capability to reduce the stress by shrinking the rest of the film along the perpendicular direction of stretching, the film cannot release the tensile stress under biaxial strain, accounting for the lower critical strain of crack.

While the low stretchability of the flat P3HT film is attributed to the high crystallinity^20^, the low stretchability of the flat RP–T50–COOH can be ascribed by its high *T*_g_ (107 °C)^29^. By contrast, the wrinkled RP–T50–COOH film can exhibit significantly enhanced stretchability (albeit the high *T*_g_ of RP–T50–COOH) because the wrinkles are physically expanded along the applied strain direction without much impact to the morphology/structure of RP–T50–COOH films under lower tensile strain (Fig. 4e). However, continuous increase of the tensile strain will eventually rupture the polymers, first elongating the entangled polymer chains in the amorphous region and then the ordered region, and finally leading to the cracking of the film^30^. We also measured the resistance change versus the applied uniaxial strain for various wrinkle wavelengths (Fig. 4f). As the wavelength of the wrinkle rises, the stretchability of the film increases^30^. Furthermore, we found that the wrinkled RP–T50–COOH film with an average wrinkle wavelength of 1.66 µm exhibits impressive mechanical robustness under uniaxial cyclic test up to 1000 repeated cycles (strain = 0 % → 50 % → 0 %) (Fig. 4g).

### Stretchable multifunctional sensors

**Fig. 5:**
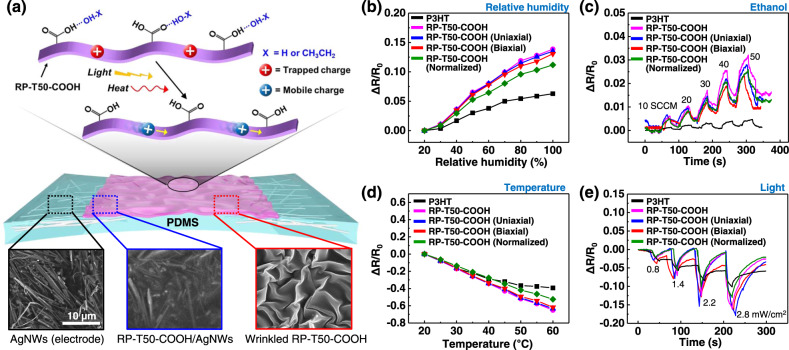
Stretchable multifunctional sensor and sensing performance. **a** Schematic illustration of the structure and working mechanisms for the stretchable sensor. Inset: SEM images of the surface morphologies on each sensor part. Resistance changes as a function of relative humidity (**b**), ethanol flow (**c**), temperature, (**d**) and light intensity (**e**) of flat P3HT and wrinkled RP–T50–COOH films. Wrinkled RP–T50–COOH films under pristine state, uniaxially stretched state (50%), and biaxially stretched state (30%) are compared. In addition, normalized resistance changes of RP–T50–COOH film under pristine state (i.e., normalized by the dimensionless factor of the initial surface area over the wrinkled surface area) is also compared.

With a high SCLC mobility (i.e., 7.46 ± 4.67 × 10^−3^ cm^2^/V·s), high stability of RP–T50–COOH and the impressive stretchability of the wrinkled RP–T50–COOH film on a PDMS substrate, we fabricated stretchable sensors, together with P3HT-based ones for comparison. As shown in Fig. 5a, the stretchable sensor was constructed by integrating wrinkled RP–T50–COOH film as the active layer and partially embedded silver nanowires (AgNWs) in PDMS as the two electrodes in series^31,32^. The detailed fabrication procedure is described in the Supplementary Information.

Firstly, we investigated sensing performance of wrinkled RP–T50–COOH and P3HT without applying strains by measuring the resistance changes as a function of relative humidity or ethanol flow. The adsorption of water or ethanol molecules to CP films can lower the number of major charge carriers in the CP films and therefore result in the increase of the electrical resistance^33^. The wrinkled RP–T50–COOH based stretchable sensor shows much higher sensitivity to humidity than P3HT sensors (Fig. 5b). Moreover, the wrinkled RP–T50–COOH also can easily detect the change of the ethanol flow from the rate of 10 standard cubic centimeters per minute (SCCM) to 50 SCCM, while P3HT only shows negligible sensitivity (Fig. 5c). This is likely because the presence of carboxylic acids in RP–T50–COOH allows the sensing of water or ethanol molecules by capturing these analyte molecules via hydrogen bonding and modifying the electrical resistance^34,35^. By contrast, P3HT does not contain functionalities that can interact with water or ethanol, thus shows lower sensitivity. However, the ethanol sensing signals of RP–T50–COOH-based sensors did not recover to their initial values even after the removal of the ethanol flow (Fig. 5c). This observation likely results from the remaining ethanol molecules on the surface of wrinkled RP–T50–COOH film (due to physical adsorption or hydrogen bonding with carboxylic acids in RP–T50–COOH) even after the removal of the ethanol flow. We believe that introducing additional nitrogen purging or slight heating to further remove the remaining ethanol molecules on the RP–T50–COOH could remove such hysteresis.

Furthermore, CP films can sense temperature and light since these external factors can change the charge carrier concentration and also cause the escape of charge carriers from traps in the CP film, leading to lower resistance upon exposure to these external factors^36–41^. Again, the wrinkled RP–T50–COOH-based sensor performs noticeably better than the P3HT-based counterpart. For example, the wrinkled RP–T50–COOH-based sensor can sense the temperature between 20 °C to 60 °C with an almost linear correlation, while the P3HT-based sensor shows lower sensitivity (Fig. 5d). This can be attributed to the higher charge mobility of RP–T50–COOH than that of P3HT. We also characterized the light sensing performance by varying the intensity of the light source (iPhone 12 pro, Apple^©^) (Fig. 5e). When the light intensity increased from 0.8 to 2.8 mW/cm^2^ (in on-off cycles), the resistances (when the light was on) were gradually decreased with the light intensity due to the photoexcitation process that creates more mobile charges^42^. Again, the light sensitivity of RP–T50–COOH-based sensor is much higher than that of P3HT-based one, which can also be explained by a higher charge mobility of RP–T50–COOH. The resistance of RP–T50–COOH-based sensors nearly recovered to their initial values except that of the biaxially stretched RP–T50–COOH based sensor. This could be attributed to the limited charge transport in the biaxially stretched RP–T50–COOH film due to the morphological changes induced by the biaxial strain (30%). Please note that the resistance of P3HT-based sensor shows more significant hysteresis likely due to the lower charge mobility of P3HT than that of RP–T50–COOH. We believe that the resistance of the biaxially stretched RP–T50–COOH sensor would be able to recover to its initial value if sufficient recovery time were given after the light off.

Finally, to evaluate the specific performance of the wrinkled RP–T50–COOH-based sensor under stretching conditions, we measured every output sensing signal under both uniaxial strain of 50% and biaxial strain of 30%. Pleasingly, the wrinkled RP–T50–COOH-based sensors maintained the sensing performance without severe sensitivity degradation under the tensile strains (Fig. 5b–e)^30,43^. In addition, we normalized every data obtained from the wrinkled RP–T50–COOH sensor by the dimensionless factor of the initial surface area over the wrinkled surface area. Though the sensor performance of wrinkled RP–T50–COOH was reduced slightly by such normalization, the sensitivity of every sensor was still much higher than the sensitivity of P3HT-based sensor. Therefore, it is clear that the great performance of RP–T50–COOH originated from the intrinsic properties of the material rather than the increased surface area.

In summary, the newly synthesized random copolymer RP-T50 by copolymerizing the thiophene monomer bearing a TCS (50 mol%) and the unsubstituted thiophene monomer (50 mol%) has demonstrated rather unique combination of high mobility, excellent stability and impressive stretchability with its wrinkled film after thermocleavage of alkyl chains. The unsubstituted thiophene units in RP-T50 can mitigate the steric hindrance of the bulky TCSs, leading to enhanced polymer chain ordering (before cleavage of the alkyl chains), and enable less disruption to the pre-annealing morphology of the RP-T50 film during thermocleavage of alkyl chains (due to less TCS) compared to P3ET containing 100 mol% TCSs. Therefore, the RP-T50 film annealed at 150 °C (i.e., RP–T50–COOH film) exhibits a high degree of π–π stacking, resulting in a higher charge mobility than P3HT; this is the first observation that a CP containing TCSs shows higher mobility than that of an identical CP with a conventional alkyl side chains. In addition, the resulting RP–T50–COOH film shows great stability against chloroform wash and under harsh conditions (100 °C, in air and under continuous ambient light for 24 h). Furthermore, the enhanced rigidity of RP–T50–COOH allows spontaneous wrinkle formation in the RP–T50–COOH film coated on a stretchable PDMS substrate, and the wrinkled structure affords considerable improvement in the stretchability under both uniaxial and biaxial tensile strains. Finally, the wrinkled RP–T50–COOH-based stretchable sensors exhibit great sensing performance toward various external stimuli such as humidity, ethanol, temperature and light even under 50% uniaxial strain and 30% biaxial strain.

## Methods

### General experimental details

All chemicals were purchased from commercial source (Sigma-Aldrich, Fisher, Acros, etc.) and were used as received except when specified. RP-TCS50 was prepared according to the previously reported procedures^4^. Size exclusion chromatography (SEC) was performed on a Waters 2695 separations module liquid chromatograph equipped with two Agilent Resipore columns (PL1113-6300) maintained at 35 °C and a Waters 2412 refractive index detector at room temperature. THF was used as the mobile phase at a flow rate of 1.0 mL/min. Molecular weight and dispersity data are reported relative to 580−200,000 g/mol poly(styrene) standards. ^1^H nuclear magnetic resonance (NMR) measurements were recorded with Bruker DRX spectrometers (500 MHz). Samples were analyzed with a Q Exactive HF-X (ThermoFisher, Bremen, Germany) mass spectrometer. Samples were introduced via a heated electrospray ionization (HESI) source at a flow rate of 10 µL/min. Xcalibur (ThermoFisher, Breman, Germany) was used to analyze the data. Molecular formula assignments were determined with Molecular Formula Calculator (v 1.2.3). UV−visible absorption spectra were obtained with a Shimadzu UV-2600 spectrophotometer. For determination of solubility, saturated solutions of the polymers were prepared and subsequently diluted to obtain the optical density. Then, the solubility was calculated by using Beer−Lambert law and comparing the optical density to known concentrations. IR spectra were performed on a Bruker Optics Hyperion 1000 with Tensor 27. The film thicknesses were recorded by a profilometer (Alpha-Step 200, Tencor Instruments). Differential scanning calorimetric measurements were performed on a TA Instruments Discovery DSC at a heating and cooling rate of 10 °C/min. Thermal gravimetric analysis (TGA) was performed on a TA Instruments Q5000 Thermogravimetric Analyzer under N_2_ atmosphere. The morphology of each sample was measured by using field emission scanning electron microscopy (FESEM) (FEI Quanta 3D FEG) and optical microscopy (OM) (Nikon ECLIPSE LV150N). The grazing incidence wide-angle X-ray scattering (GIWAXS) measurement was performed at a synchrotron radiation facility on the beamline 9 A at Pohang Accelerator Laboratory (PAL), Korea. GIWAXS experiments were carried in a vacuum chamber with a sample-to-detector distance of 212 mm. The incidence angle for the X-ray was set to 0.14°. The electrical resistances of the stretchable thin film and sensor were characterized by a digital multimeter (Keysight DAQ970A). For the investigation of light sensing performance, the light was generated by a commercial mobile phone (Apple iPhone 12 Pro). The light intensity was measured using an optical multimeter (Newport 1919-R).

### Synthesis of P3HT, P3ET and RP-T50

The detailed synthetic procedures are provided in the Supplementary Information.

### SCLC mobility measurement

SCLC mobility was acquired through the hole-only devices with a configuration of ITO/PEDOT:PSS/active layer/MoO_3_/Al. The experimental dark current densities *J* were measured by Keithley 2400. The applied voltage *V* was corrected from the voltage drop *V*_rs_ due to the series resistance and contact resistance, which were found from a reference device without the active layer, and the build-in potential, which are estimated from the *V*_OC_ of corresponding hole-only devices under 1 sun condition. From the plots of J 0.5 vs V, hole mobilities of polymers were deduced from the Mott−Gurneys law:

$$J=\frac{9}{8}{\varepsilon }_{r}{\varepsilon }_{0}{\mu }_{h}\frac{{V}^{2}}{{L}^{3}}$$

where ε_0_ is the permittivity of free space, ε_r_ is the dielectric constant of the polymer which is assumed to be around 3, μ_h_ is the hole mobility, *V* is the voltage drop across the device, and *L* is the film thickness of the active layer.

## Supplementary information

Supplementary Information

## Data Availability

All data supporting the findings of this study are provided within this article and its Supplementary Information.
