# Supplementary material for: Integrating charge mobility, stability and stretchability within conjugated polymer films for stretchable multifunctional sensors
Source: Nat Commun. 2022 May 18;13:2739. doi: 10.1038/s41467-022-30361-0 (PMC9117230; doi:10.1038/s41467-022-30361-0)
Supplement: Supplementary file 1 — Supplementary Information [file 41467_2022_30361_MOESM1_ESM.pdf]

Supplementary Information

Integrating Charge Mobility, Stability and Stretchability within Conjugated Polymer Films for Stretchable Multifunctional Sensors

Sung Yun Son [1,2], Giwon Lee[3,4], Hongyu Wang[4], Stephanie Samson[5], Qingshan Wei[3], Yong Zhu[4]* and Wei You[1]**

[1]Department of Chemistry, University of North Carolina at Chapel Hill, Chapel Hill, NC 27599, USA

[2]Department of Chemistry, Kwangwoon University, Seoul 01897, Republic of Korea

[3]Department of Chemical and Biomolecular Engineering, North Carolina State University, Raleigh, NC 27695, USA

[4]Department of Mechanical and Aerospace Engineering, North Carolina State University, Raleigh, NC 27695, USA

[5]Department of Applied Physical Sciences, University of North Carolina at Chapel Hill, Chapel Hill, NC 27599, USA

These authors contributed equally: Sung Yun Son, Giwon Lee

*Corresponding authors

*E-mail: qwei3@ncsu.edu, yzhu7@ncsu.edu and wyou@unc.edu

Supplementary Methods

- Detailed synthetic procedures

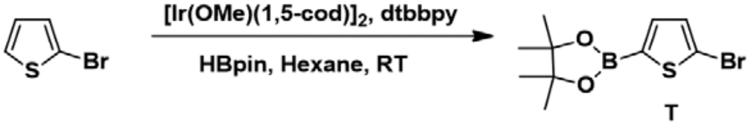

2-Bromo-5-(4,4,5,5-tetramethyl-1,3,2-dioxaborolan-2-yl)thiophene (**T**). In a glove box under N_2 atmosphere, a 50 mL round bottom flask was charged with pinacolborane (HBpin) (1.28 g, 10 mmol), di-μ-methoxobis(1,5-cyclooctadiene)diiridium (49.7 mg, 0.075 mmol) and anhydrous hexane (4 mL). To the reaction mixture, 4,4'-Bis(di-*t*-butyl)-2,2'-bipyridine (dtbbpy) (38.9 mg, 0.145 mmol) in anhydrous hexane (4 mL) was added, then the reaction mixture was stirred for 15 min. 2-Bromo-thiophene (815 mg, 5 mmol) dissolved in anhydrous hexane (4 mL) was added to the reaction mixture slowly (H_2 gas evolves). The solution was stirred overnight in the glove box. The reaction was taken out of the glove box and loaded directly onto silica gel, then eluted with hexane:DCM = 10:1. The product was collected as a pale yellow oil (1.28 g, 88.6 %).

^{1}H NMR (500 MHz, $CDCl_3$, δ): 7.37 (d, J = 3.7 Hz, 1H), 7.10 (d, J = 3.7 Hz, 1H), 1.33 (s, 12H).

^{13}C NMR (500 MHz, $CDCl_3$, δ): 137.93, 131.70, 119.76, 84.67, 25.07

Mass spectroscopy: $[M+H]^+$ $C_{10}H_{15}O_2SBBr$; m/z = 289.00622 ; mass error = -0.3 ppm

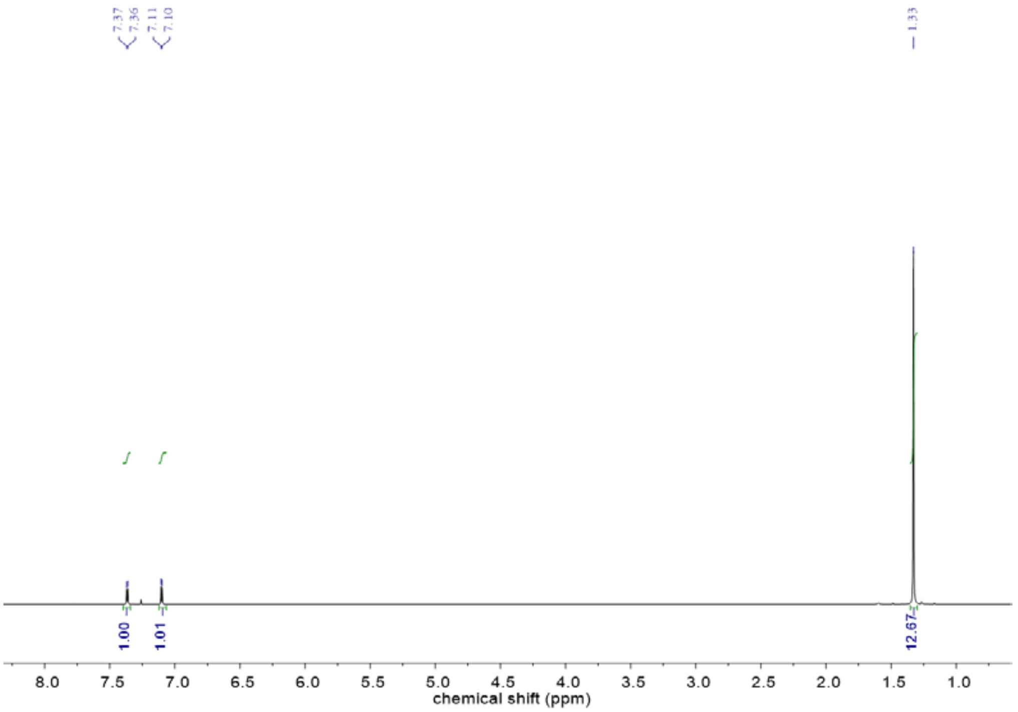

^{1}H NMR spectrum of T

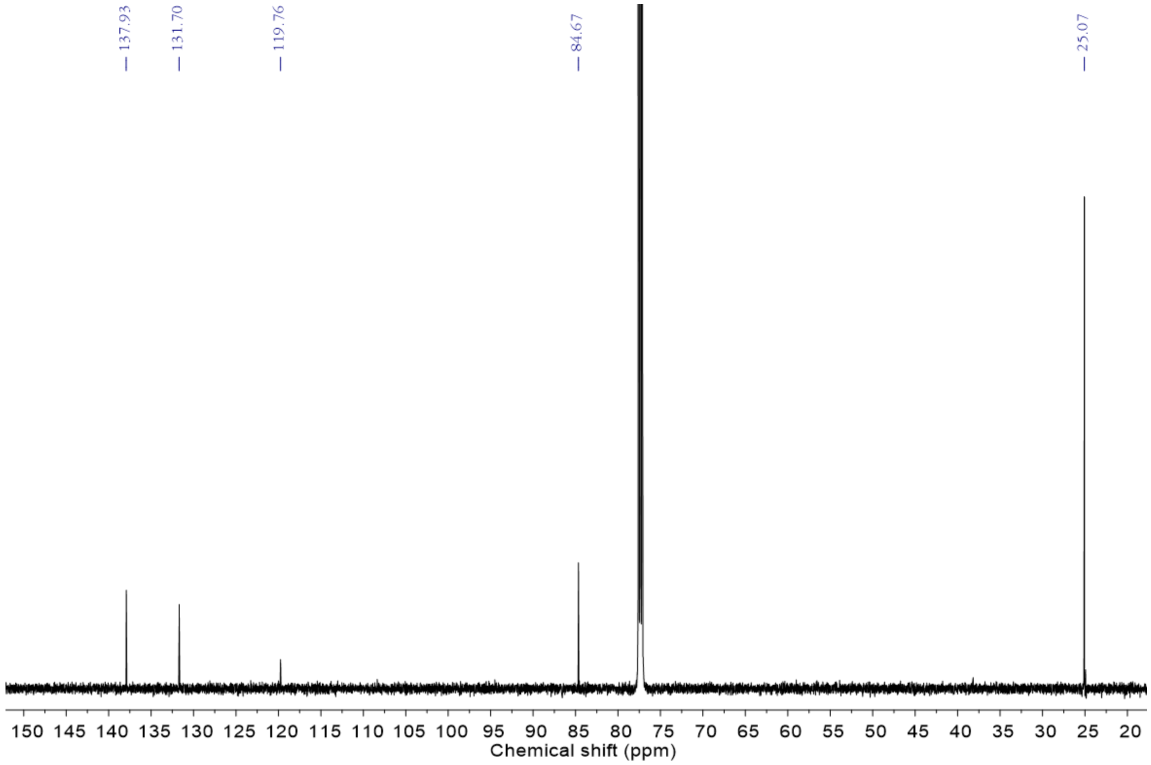

^{13}C NMR of T

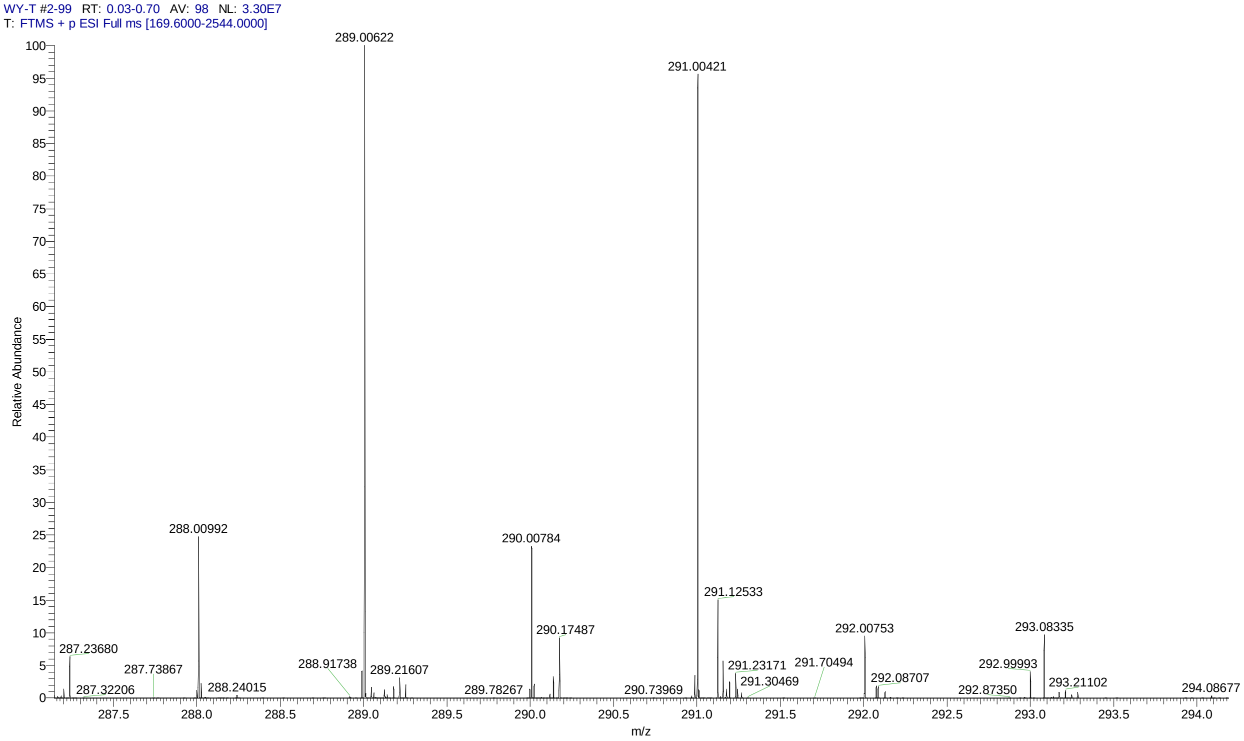

High resolution mass spectrum of T

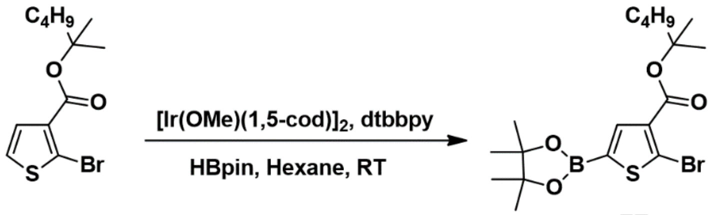

ET

2-Methylhexan-2-yl 2-bromo-5-(4,4,5,5-tetramethyl-1,3,2-dioxaborolan-2-yl)thiophene-3-carboxylate (**ET**). **ET** was synthesized according to the previously reported procedures [1] In a glove box under N_2 atmosphere, a 40 mL scintillation vial was charged with pinacolborane (HBpin) (1.28 g, 10 mmol), di-μ-methoxobis(1,5-cyclooctadiene)diiridium (49.7 mg, 0.075 mmol) and dry hexane (4 mL). To the reaction mixture, 4,4'-Bis(di-*t*-butyl)-2,2'-bipyridine (dtbbpy) (38.9 mg, 0.145 mmol) in hexane (4 mL) was added, then the reaction mixture was stirred for 15 min. 2-Methylhexan-2-yl 2-bromothiophene-3-carboxylate (1.53 g, 5 mmol) dissolved in hexane (4 mL) was added to the reaction mixture slowly (H_2 gas evolves). The solution was stirred overnight in the glove box. The reaction was taken out of the glove box and loaded directly onto silica gel, then eluted with hexane:DCM = 1:1. The product was collected as a clear oil and slowly solidified after drying *in vacuo* to afford white powder (1.85 g, 4.3 mmol, 86 %).

^{1}H NMR (500 MHz, $CDCl_3$, ppm): δ = 7.80 (s, 1H), 1.91 (t, J = 7.8 Hz, 2H), 1.57 (s, 6H), 1.32-1.40 (m, 16H), 0.93 (t, J = 7.0 Hz, 3H).

^{13}C NMR (500 MHz, $CDCl_3$, δ): 161.39, 139.41, 134.07, 125.66, 84.95, 84.69, 40.63, 26.66, 25.06, 23.35, 14.44.

Mass spectroscopy: $[M+H]^+$ $C_{18}H_{29}O_4SBBr$; m/z = 431.10541 ; mass error = -0.3 ppm

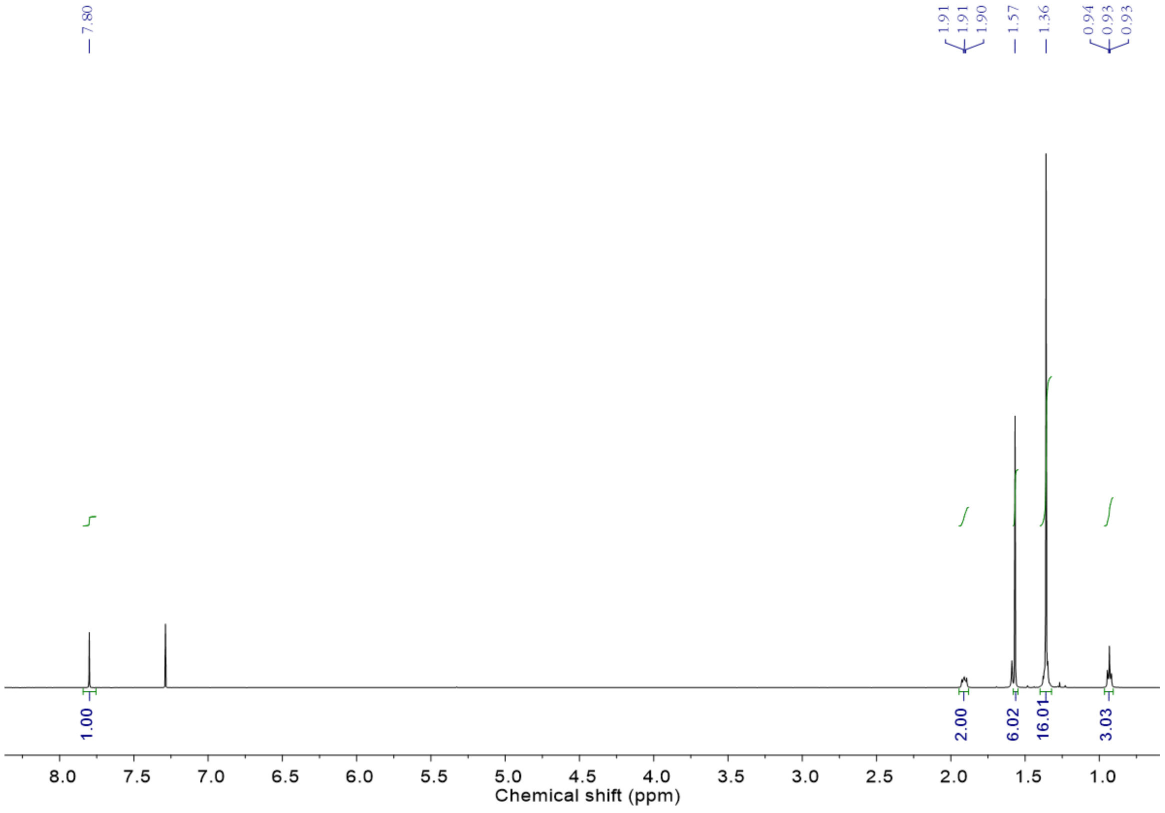

^{1}H NMR of ET

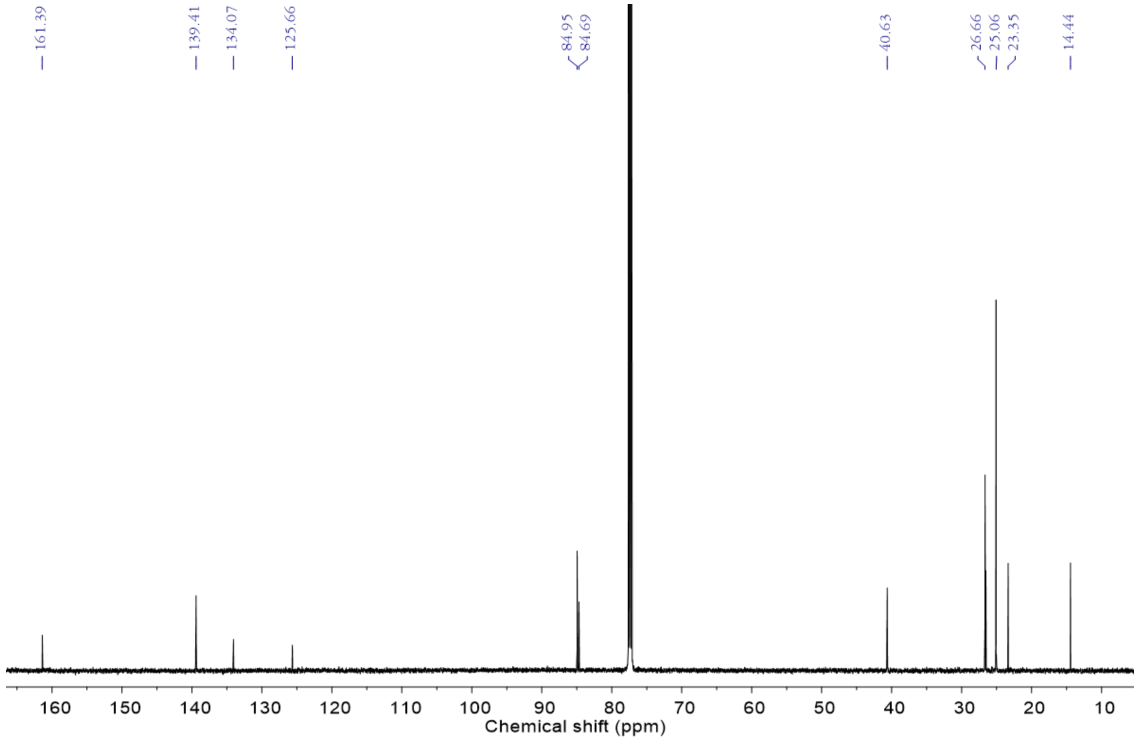

^{13}C NMR of ET

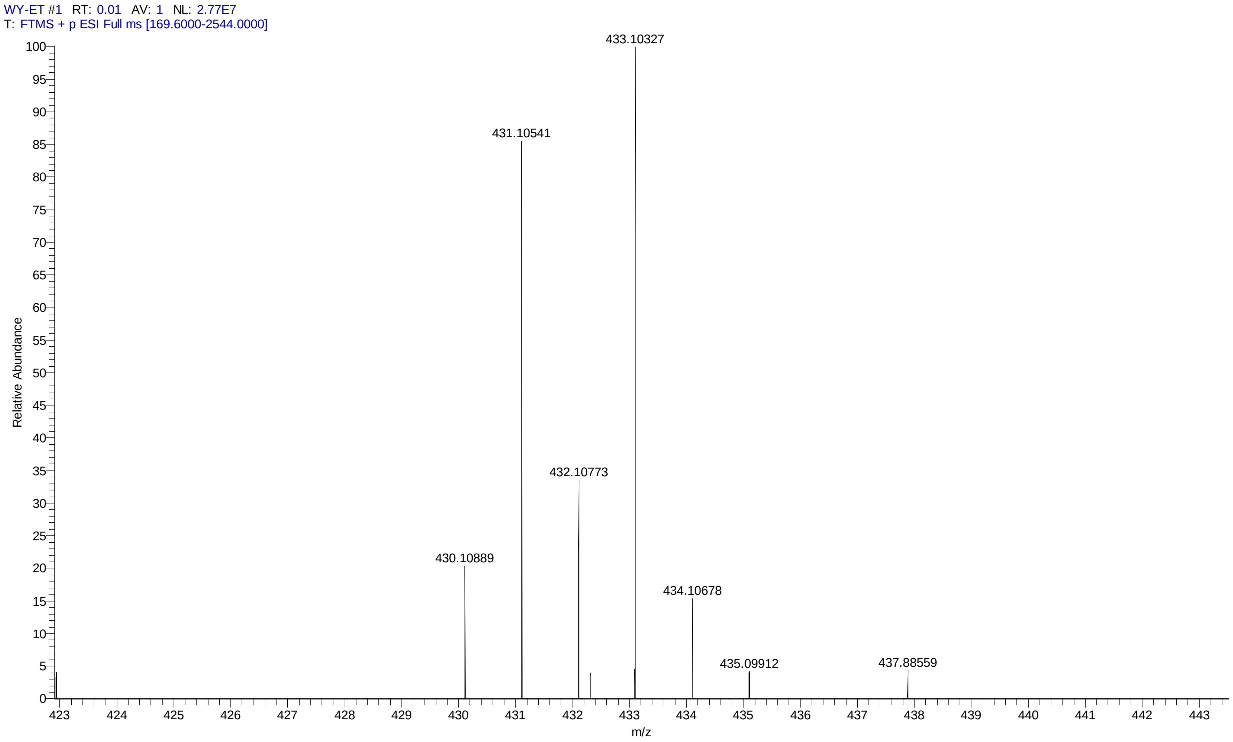

High resolution mass spectrum of ET

Suzuki polycondensation for RP-Ts synthesis. In a 20 mL scintillation vial charged with $Ni(PPh_3)IPrCl_2$ (7.8 mg, 0.01 mmol), $K_3PO_4 \cdot H_2O$ (132 mg, 0.575 mmol), **ET** and **T** were added in the appropriate monomer feed ratio at 0.5 mmol total monomer scale (e.g., 108 mg (0.25 mmol) of **ET** and 72.3 mg (0.25 mmol) of **T** for RP-T50 synthesis). After the scintillation vial was evacuated and refilled three times with argon, anhydrous THF (4 mL) and degassed H_2O (0.1 mL) were injected. Then, the reaction mixture was stirred at 50 °C. After 3 h of stirring, the reaction was quenched with 5 M methanolic HCl. The precipitate was filtered and then purified by Soxhlet extraction with methanol, hexane, and chloroform. The chloroform fraction was concentrated under reduced pressure, and poured into methanol. The polymer was collected via filtration, which was then dried in a vacuum oven for 24 h at room temperature.

Suzuki polycondensation for P3HT synthesis. P3HT was synthesized according to the previously reported procedures [1] In a 20 mL scintillation vial charged with $Ni(dppp)Cl_2$ (10.8 mg, 0.02 mmol), $K_3PO_4 \cdot H_2O$ (264.8 mg, 1.15 mmol), **HT** (373.2 mg 1 mmol) was added. After the vial was evacuated and refilled three times with argon, anhydrous THF (4 mL) and degassed H_2O (0.1 mL) were injected. Then, the reaction mixture was stirred at 50 °C. After 3 h of stirring, the

reaction was quenched with 5 M methanolic HCl. The precipitate was filtered and then purified by Soxhlet extraction with methanol, hexane, and chloroform. The chloroform fraction was concentrated under reduced pressure, and poured into methanol. The polymer was collected via filtration, which was then dried in a vacuum oven for 24 h at room temperature.

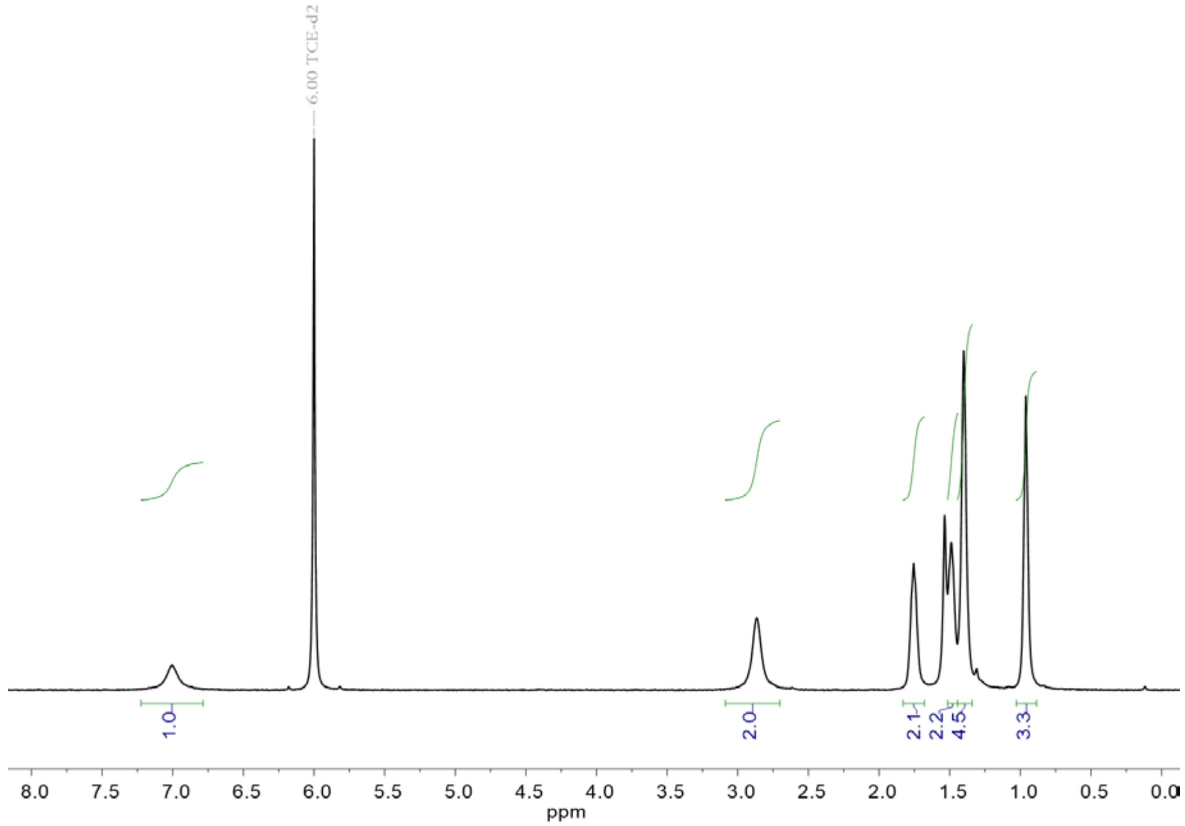

^{1}H NMR spectrum of P3HT. 1,1,2,2-Tetrachloroethane_d_2 was used as a solvent. The spectrum was obtained at 60 °C

Suzuki polycondensation for P3ET synthesis. P3ET was synthesized according to the previously reported procedures [1] In a 20 mL scintillation vial charged with $Ni(PPh_3)IPrCl_2$ (15.6 mg, 0.02 mmol), $K_3PO_4 \cdot H_2O$ (264.8 mg, 1.15 mmol), **ET** (431.2 mg 1 mmol) was added. After the vial was evacuated and refilled three times with argon, anhydrous THF (4 mL) and degassed H_2O (0.1 mL) were injected. Then, the reaction mixture was stirred at 50 °C. After 3 h of stirring, the reaction was quenched with 5 M methanolic HCl. The precipitate was filtered and then purified by Soxhlet extraction with methanol, hexane, and chloroform. The chloroform fraction was concentrated under reduced pressure, and poured into methanol. The polymer was collected via filtration, which was then dried in a vacuum oven for 24 h at room temperature.

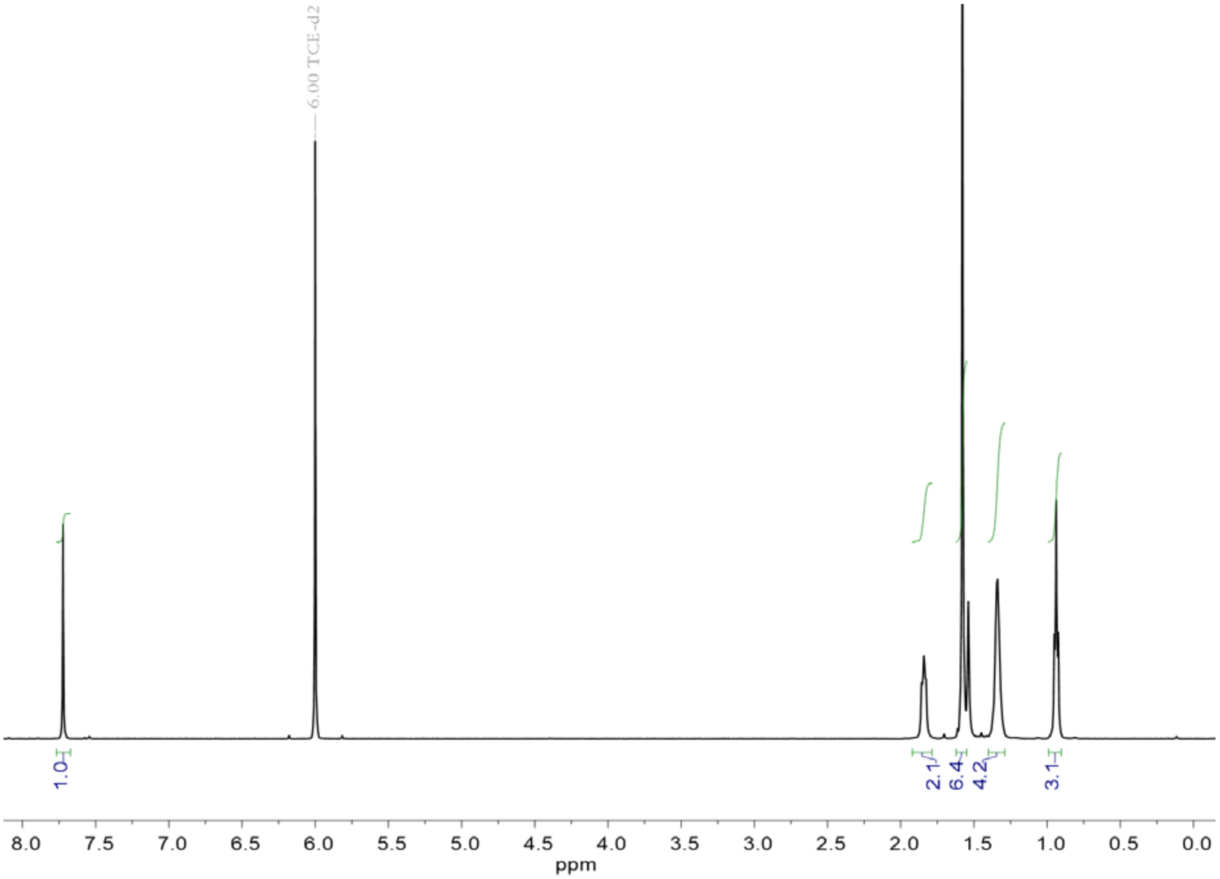

^{1}H NMR spectrum of P3ET. 1,1,2,2-Tetrachloroethane_d$_2$ was used as a solvent. The spectrum was obtained at 60 °C

- Preparation of stretchable sensors

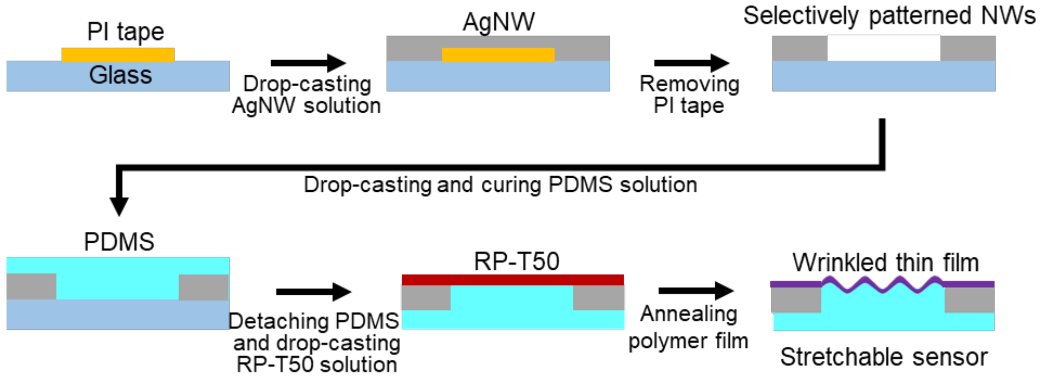

PI tape was attached on glass slide for selective patterning of electrodes. AgNW solution (solvent: water, 20 mg/ml) was drop-casted on the glass substrate. AgNWs were synthesized with the

polyol method.[2,3] After evaporation of the solution, the PI tape was removed and selectively patterned NWs were formed on the glass. PDMS solution was poured on the substrate. The PDMS film was prepared by mixing a PDMS prepolymer and a curing agent (Sylgard 184, Dow Corning, weight ratio 20:1). When PDMS was fully cured, we detached PDMS from the glass. In this stage, partially embedded AgNWs in PDMS were successfully obtained. With this sensor patch, RP-T50 solution (solvent: chloroform, 1 mg/ml) was drop-casted on the patch. After evaporation of the solution, the polymer film was annealed with 150 °C for 12 h and cooled in room temperature. As a result, the stretchable sensor was developed with surface wrinkling of the polymer film as a sensing layer.

- Modulus calculation before and after side chain cleavage.

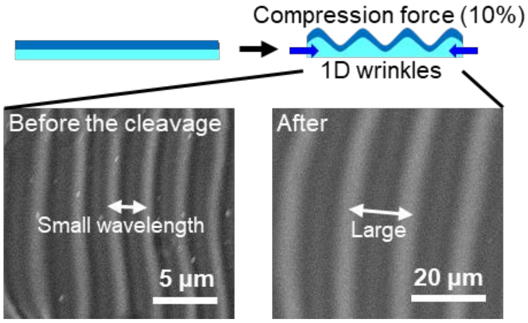

1D wrinkles were intentionally generated by using the compression force of 10% for measuring the modulus of each polymer film before and after side chain cleavage (**Figure shown above**). Based on the buckling theory, the modulus of thin film ($\bar{E}_f$) is easily calculated by the wavelength (λ) of the wrinkle, $\bar{E}_f = 3\bar{E}_s(\frac{\lambda}{2\pi h})^3$.[4] Here, h is the thickness of the thin film and subscript f and s denote film and substrate, respectively. With this method, we repeated 3 times with 3 individual samples for consistency of our results. By applying the compression force, the wavelength of the film increased from 2.3 ~ 4.9 μm (before) to 14.5 ~ 18.8 μm. The thickness of the film was different from 0.80 ~ 0.90 μm (before) to 0.50 ~ 0.59 μm (after). Therefore, according to the equation, the modulus of the thin film was calculated before the cleavage (2.57 ~ 18.7 MPa) and after (2.64 ~ 4.52 GPa). This result means that the stiffness of the thin film

could be transformed from soft state to hard one after the cleavage.

Sample #	Before cleavage			After cleavage		
	Wavelength (μm)	Thickness (μm)	Young's modulus (MPa)	Wavelength (μm)	Thickness (μm)	Young's modulus (GPa)
1	2.8	0.83	4.22	16.7	0.51	3.79
	3.5	0.81	9.06	14.5	0.50	2.64
	2.3	0.80	2.57	17.8	0.53	4.01
2	3.3	0.84	6.56	17.2	0.53	3.70
	3.1	0.81	6.06	17.7	0.51	4.52
	2.8	0.80	4.64	16.5	0.55	2.92
3	4.5	0.90	13.5	18.5	0.57	3.70
	4.9	0.88	18.7	17.9	0.59	3.02
	4.7	0.85	18.3	18.8	0.56	4.09

Supplementary Fig. 1. ^{1}H NMR spectrum of RP-T50. 1,1,2,2-Tetrachloroethane_d$_2$ was used as a solvent. The spectrum was obtained at 70 °C.

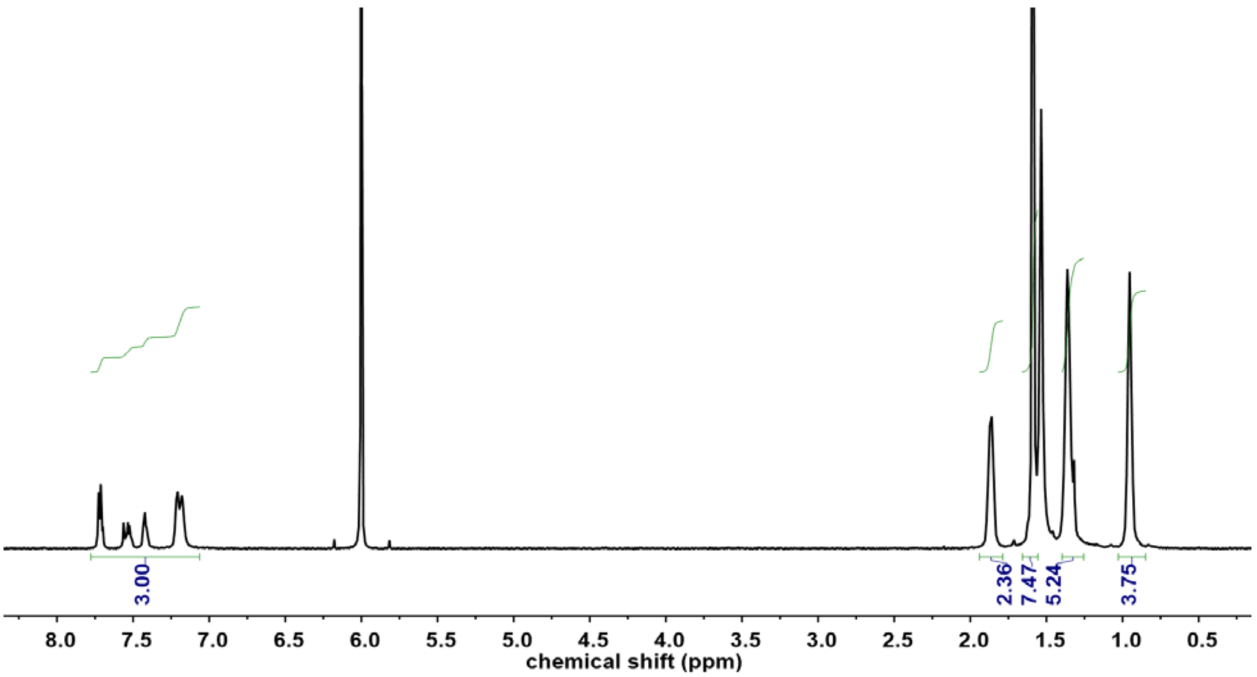

Supplementary Fig. 2. IR spectra of an as-cast RP-T50 film and 150 °C annealed RP-T50 films up to 12 h.

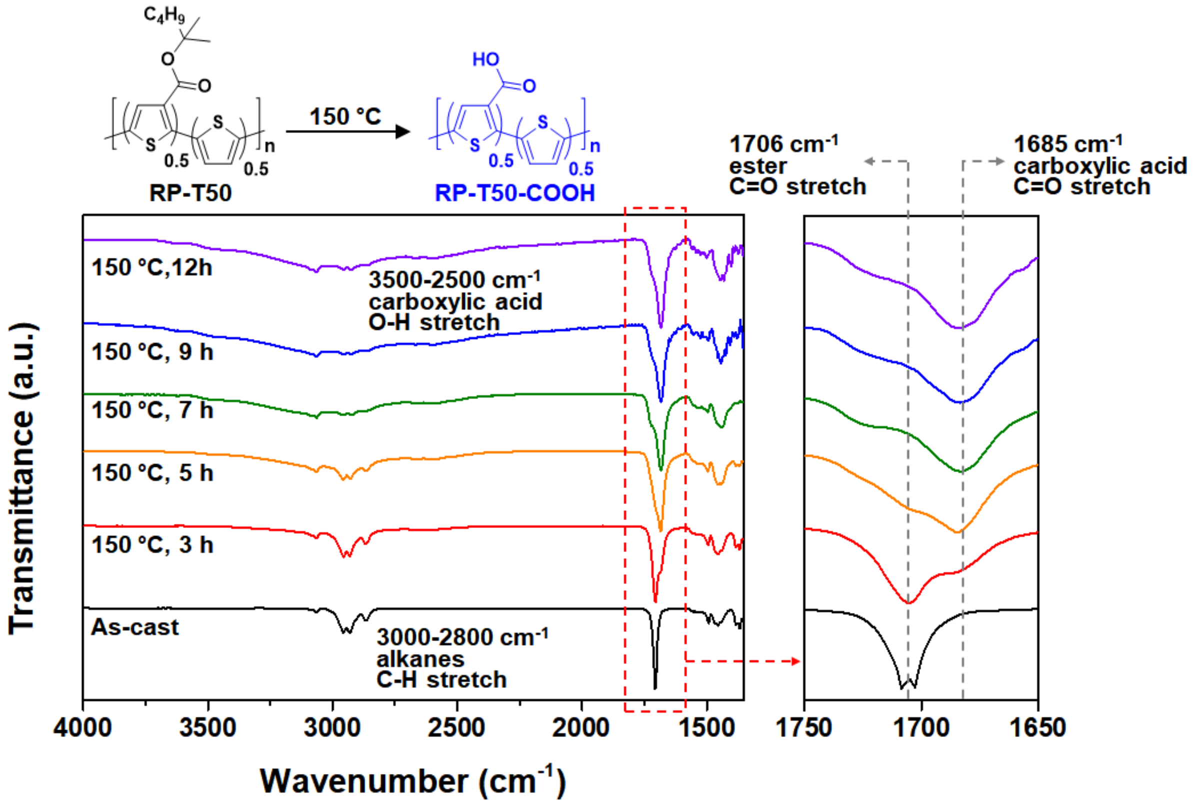

Supplementary Fig. 3. Normalized UV-vis absorption spectra of P3ET-COOH and RP-T50-COOH films.

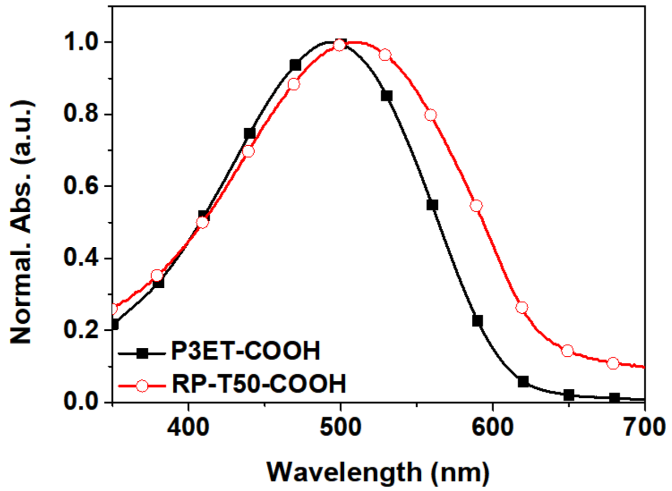

Supplementary Fig. 4. Out-of-plane spectra extracted from the GIWAXS patterns.

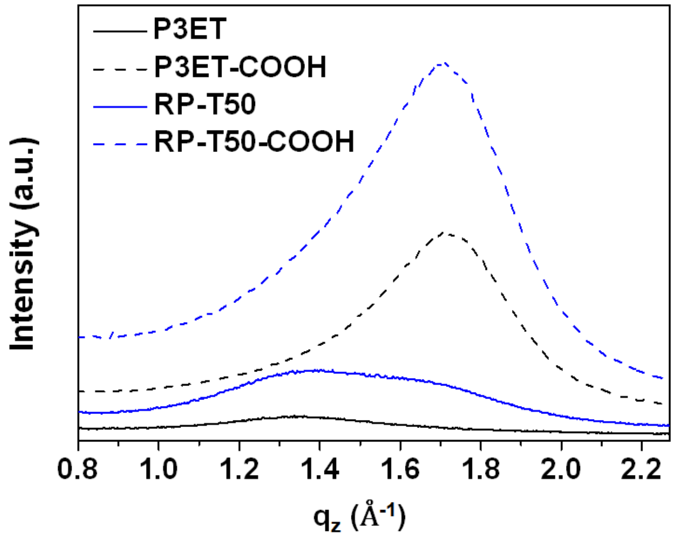

Supplementary Fig. 5. Geometrically corrected pole figures extracted from (010) scattering peaks from GIWAXS patterns for P3ET-COOH and RP-T50-COOH films.

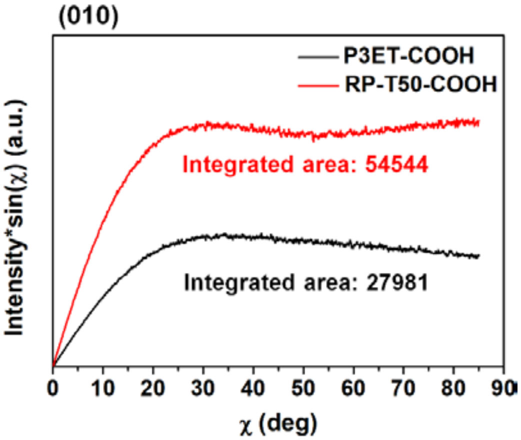

Supplementary Fig. 6. Log-log J-V curves for the annealed (150 °C, 12 h) and as-cast polymer films. The regions highlighted in red are the regions fit for SCLC; that is, where current follows the Mott-Gurney law ($J \sim V^2$). The dashed line is a guide to the eye. The inset figures are the fit line (top) and residual plot (bottom).

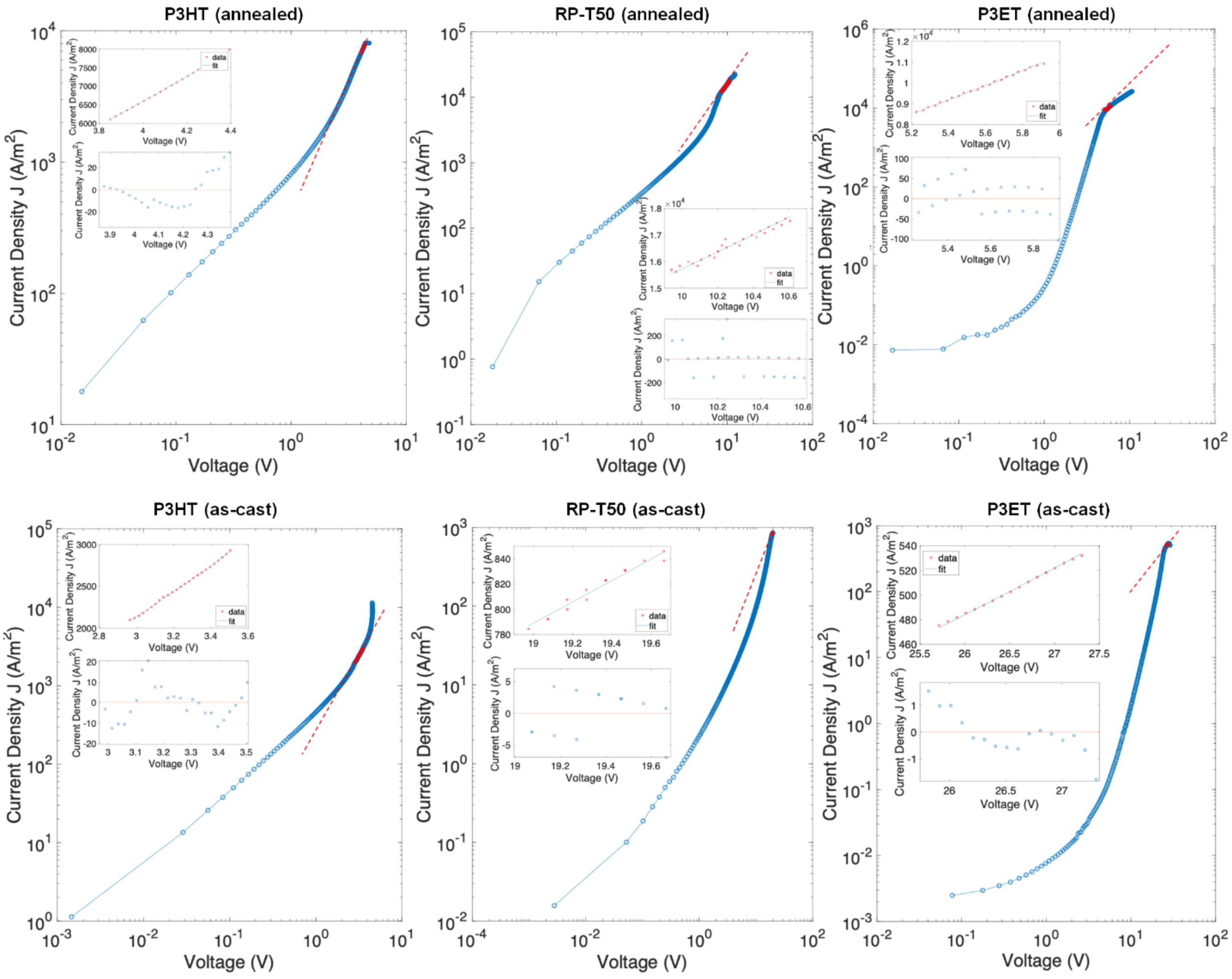

Supplementary Fig. 7. SCLC mobilities of RP-T50 and RP-TCS50 before and after annealing at 150 °C for 12 h.

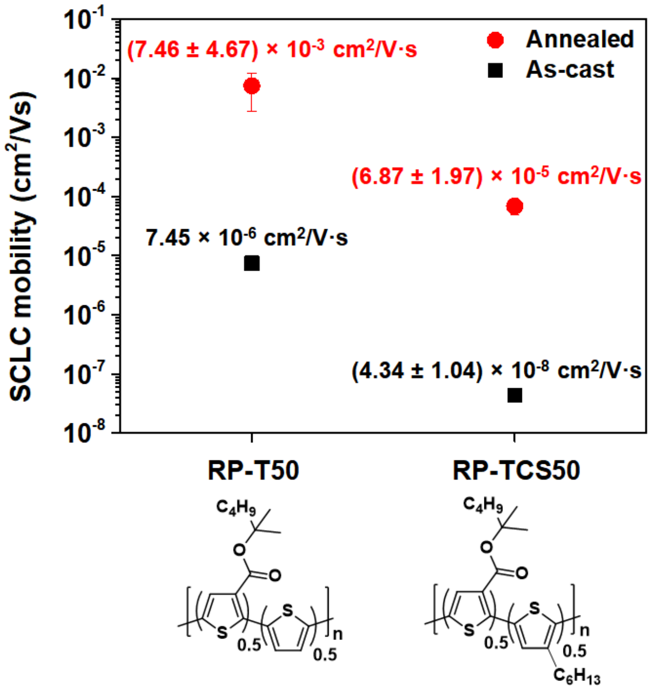

Supplementary Fig. 8. a) Schematic representation of spin-rinse with chloroform. b) as-cast and spin-rinsed P3HT films.

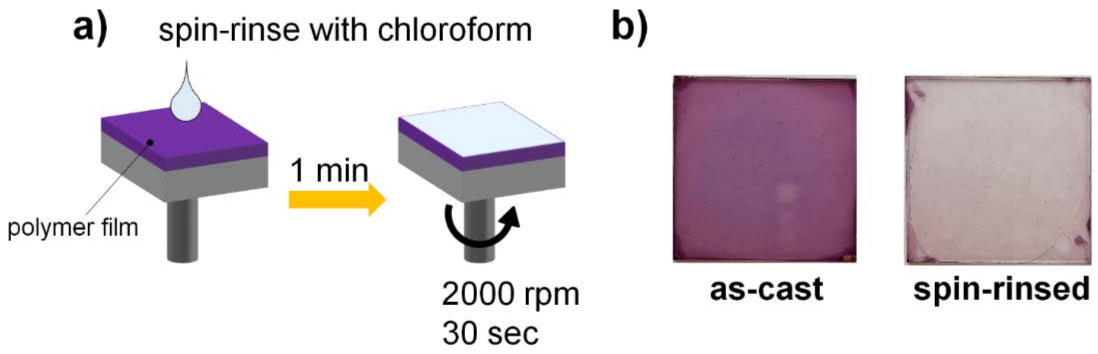

Supplementary Table 1. Theoretical weight remaining, film thickness and normalized film density of RP-T50 before and after annealing at 150 °C for 12 h.

Anneal temp. [°C]	Theoretical weight remaining after cleavage [%]	Film thickness [nm]		Average normalized thickness	Normalized film density[a]
		1st film	2nd film		
None	100	915	496	1	1
150	66	498	288	0.56	1.18

[a]Estimated using the theoretical weight remaining after cleavage, divided by the average normalized thickness

Supplementary Table 2. Charge mobilities of the polymers before and after thermal annealing at 150 °C for 12 h.

Polymer	Charge mobility ($cm^2/V \cdot s$)[a]	
	Before annealing	After annealing
P3HT	$(9.18 \pm 5.44) \times 10^{-4}$	$(6.67 \pm 2.67) \times 10^{-4}$
P3ET	$(1.76 \pm 0.01) \times 10^{-6}$	$(1.53 \pm 0.29) \times 10^{-4}$
RP-T50	7.45×10^{-6}	$(7.46 \pm 4.67) \times 10^{-3}$

[a]Obtained from 2-4 devices except RP-T50 (before annealing). The charge mobility of RP-T50 (before annealing) was obtained from one device

Reference

[1] S. Y. Son, S. Samson, S. Siddika, B. T. O'Connor, W. You, *Chem. Mater.*, **2021**, 33, 4745.
[2] Y. Sun, Y. Xia, *Adv. Mater.*, **2002**, 14, 833.
[3] Y. Zhu, Q. Qin, F. Xu, F. Fan, Y. Ding, T. Zhang, B. J. Wiley, Z. L. Wang, *Phys. Rev. B*, **2012**, 85, 045443.
[4] D. Tahk, H. H. Lee, D. Y. Khang, *Macromolecules*, **2009**, 42, 7079.
